# Chitosan oligosaccharide alleviates renal fibrosis through reducing oxidative stress damage and regulating TGF-β1/Smads pathway

**DOI:** 10.1038/s41598-022-20719-1

**Published:** 2022-11-10

**Authors:** Jun Wu, Yingtao Xu, Zikai Geng, Jianqing Zhou, Qingping Xiong, Zhimeng Xu, Hailun Li, Yun Han

**Affiliations:** 1 School of Chinese Medicine, Shandong College of Traditional Chinese Medicine, Yantai, 264199 Shandong People’s Republic of China; 2 Department of Nephrology, Affiliated Huai’an Hospital of Xuzhou Medical University, 223002, Huai’an, Jiangsu People’s Republic of China; 3 School of Integrated Chinese and Western Medicine, Binzhou Medical University, Yantai, 264003 Shandong People’s Republic of China; 4 Science and Technology Innovation Center, Guangzhou University of Chinese Medicine, Guangzhou, 510006 Guangdong People’s Republic of China; 5 Jiangsu Key Laboratory of Regional Resource Exploitation and Medicinal Research, Huaiyin Institute of Technology, Huai’an, 223003 Jiangsu People’s Republic of China; 6 Department of Food, Jiangsu Food and Pharmaceutical Science College, Huai’an, 223003 Jiangsu China

**Keywords:** Renal fibrosis, Drug development, Translational research

## Abstract

Renal fibrosis (RF) is the common pathway for a variety of chronic kidney diseases that progress to end-stage renal disease. Chitosan oligosaccharide (COS) has been identified as possessing many health functions. However, it is not clear whether COS can prevent RF. The purpose of this paper was to explore the action and mechanism of COS in alleviating RF. First, an acute unilateral ureteral obstruction operation (UUO) in male BALB/c mice was performed to induce RF, and COS or fosinopril (positive control drug) were administered for 7 consecutive days. Data from our experiments indicated that COS treatment can significantly alleviate kidney injury and decrease the levels of blood urea nitrogen (BUN) and serum creatinine (SCr) in the UUO mouse model. More importantly, our results show that COS can reduce collagen deposition and decrease the expression of fibrosis proteins, such as collagen IV, fibronectin, collagen I, α-smooth muscle actin (α-SMA) and E-cadherin, ameliorating experimental renal fibrosis in vivo. In addition, we also found that COS suppressed oxidative stress and inflammation in RF model mice. Further studies indicated that the mechanism by which COS alleviates renal fibrosis is closely related to the regulation of the TGF-β1/Smad pathway. COS has a therapeutic effect on ameliorating renal fibrosis similar to that of the positive control drug fosinopril. Taken together, COS can alleviate renal fibrosis induced by UUO by reducing oxidative stress damage and regulating the TGF-β1/Smad pathway.

## Introduction

Chronic kidney disease (CKD) is currently considered as a major public health problem^1^. CKD prevalence has been determined to be 8–16% worldwide^2^. Renal fibrosis (RF) is a clinical pathological syndrome characterized by abnormal extracellular matrix components (ECMs) stored in the kidney with many pathological causes. RF is the unifying feature of progressive renal alterations and the common pathway for a variety of CKDs progressing to end-stage renal disease (ESRD)^3^. A growing body of research indicates that RF contributes to the progressive and irreversible decline in renal function and is associated with high morbidity and mortality induced by CKD^4–6^. Therefore, it is urgent to develop an effective drug or method to prevent and treat RF, so as to slow down the ESRD process induced by CKD as much as possible. The renin–angiotensin system (RAS) is actively involved in the development of renal fibrosis, which can trigger the excessive deposition of collagen and change vascular resistance to aggravate RF. This provides an important and rational basis for using angiotensin converting enzyme inhibitors (ACE-Is) to alleviate RF^7^. Fosinopril, an ACE-I, which has been well tested in the treatment of renal injury. Previous investigations have confirmed that fosinopril can prevent inflammation and oxidative stress to inhibit fibrinoid necrosis, focal and segmental hyperplasia and interstitial infiltration in the kidney^8^. Considering the significant effect of fosinopril on RF, fosinopril was used as a positive control drug in most of the efficacy evaluation experiments on RF.

Many studies have shown that inflammation^9,10^ and oxidative stress injury^11^ have been widely accepted to play an important role in the occurrence and development of RF. Furthermore, a number of studies have also demonstrated that TGF-β/Smads signal transduction is a key pathway in progressive RF^12^. Thus, anti-inflammatory, antioxidative stress injury and TGF-β/Smads pathway regulation have been considered as important strategies for the treatment of RF.

**Figure 1:**
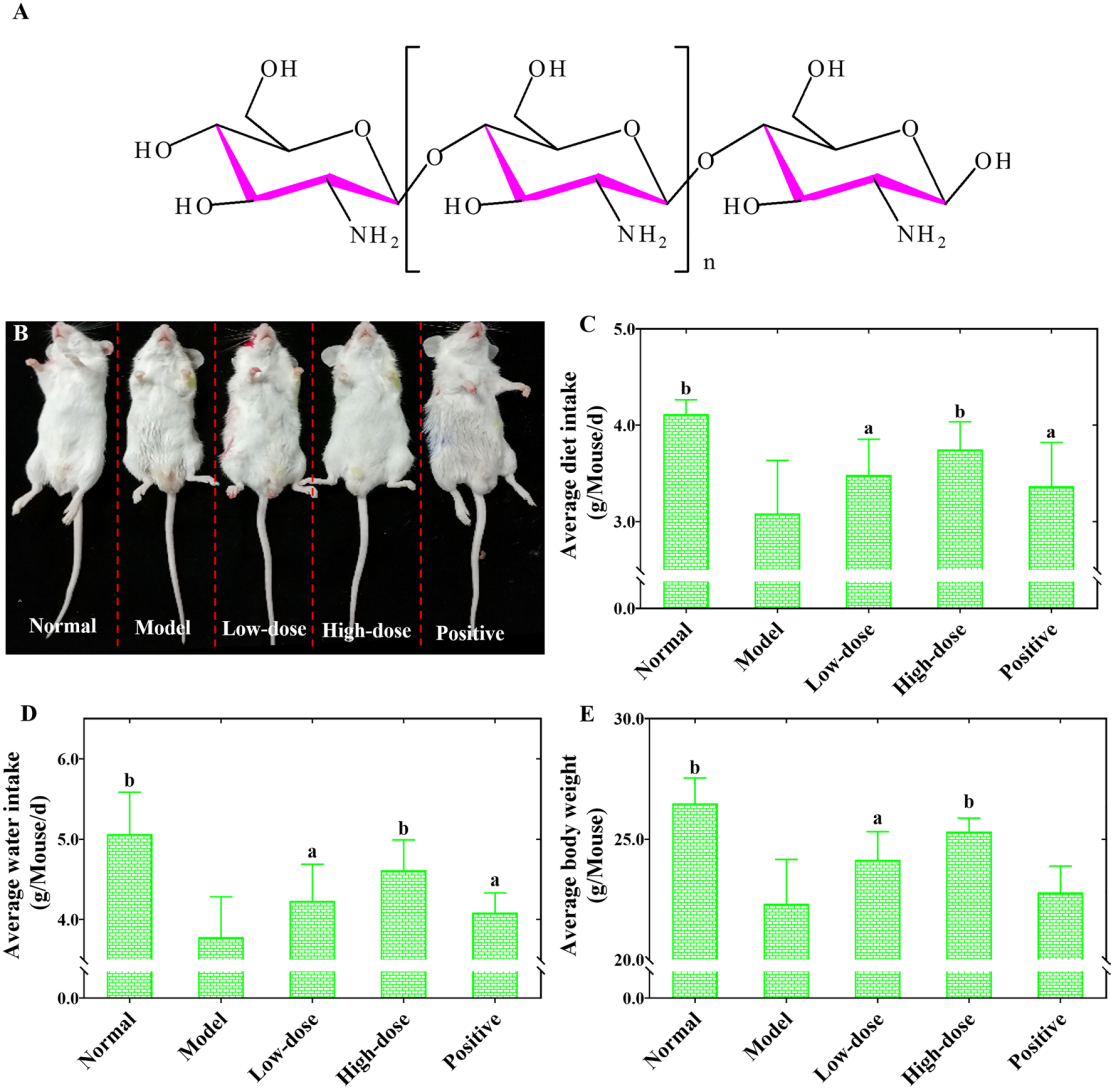
Chemical structure (**A**) and the effects of COS (**B**–**E**) on the clinical symptoms in UUO-induced RF mice. Representative somatotype pictures of mice in each group (**B**). Diet changes (**C**). Water intake changes (**D**). Body weight changes (**E**). All values are represented as mean ± SD (n = 10 in each group). Superscript letters a and b designate significant differences. ^a^*P* < 0.05 compared with the model group. ^b^*P* < 0.01 compared with the model group.

Recently, it was discovered that numerous natural polysaccharides with good antioxidant and anti-inflammatory properties and TGF-β/Smads pathway regulation have been discovered to possess potent against RF activities^13,14^, which have attracted considerable interest as potential candidates for the development of novel RF therapies. Chitosan oligosaccharide (COS), a natural oligomer polysaccharide from the depolymerized product of chitosan, consists of β-1,4-linked d-glucosamine units (Fig. 1A)^15^. Studies have shown that COS has higher water solubility, lower viscosity, and a higher rate of absorption through the intestinal epithelia than those of chitosan because it has a degree of polymerization below than 50–55 and an average molecular weight (*Mw*) of less than 10 kDa^16^. These characteristics of COS make it a preferable candidate for pharmaceutical applications. Even more encouragingly, COS has been proven to possess a variety of beneficial biological effects, including anti-inflammatory, antioxidative, antidiabetic, antihypertensive, and lipid-lowering activities^16,17^. Furthermore, there is also evidence showing that COS can regulate the TGF-β/Smads pathway to prevent and treat fibrous disease^15^.

In view of the previous findings on the biological activities of COS, it is reasonable to speculate that COS may possess a significant on anti-RF effect. However, there are still few reports, and there is a lack of relevant experimental data supporting these hypotheses. Therefore, in the present paper, we will systematically study the amelioration of RF in an acute unilateral ureteral obstruction mouse model by COS supplementation at the present paper, and further reveal its underlying mechanism.

## Results

### COS alleviated the clinical symptoms in UUO-induced RF mice

In the course of the experiment, it was found that the mice from the normal group subjected to the sham operation still had glossy fur (Fig. 1B), a good mental state, a more agile reaction, flexible movement, no obvious reduction in food and water intake (Fig. 1C,D), and a gradual increase in body weight (Fig. 1E). Compared with the normal group, the mice in the model group showed a series of pathological symptoms as the experiment progressed including a gradually worsening mental state, dull reactions, dark yellow and dull hair (Fig. 1B), significantly reduced movement, prominently decreased food and water intake (Fig. 1C,D), and markedly slower body weight gain (Fig. 1E). However, these RF symptoms in the model group were significantly improved after fosinopril (except for body weight) and COS intervention (Fig. 1). More importantly, these symptoms, especially body weight and food and water intake, showed a significant dose–response relationship in the COS intervention group. The results fully implied that COS could alleviate the clinical symptoms in UUO-induced RF.

### COS mitigated the swelling and weight of the obstructed kidney in UUO-induced RF mice

**Figure 2:**
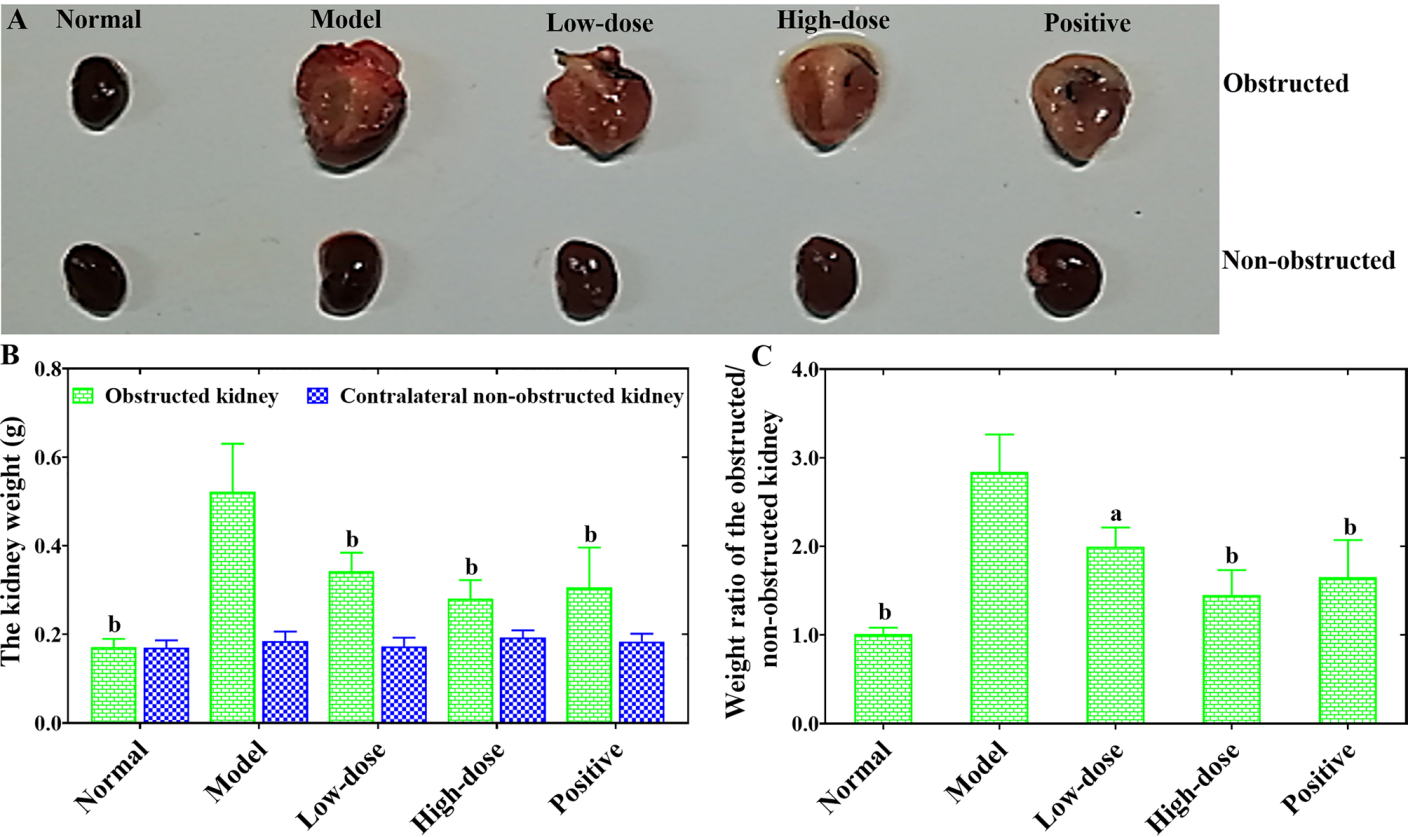
The effects of COS on the swelling and weight of the obstructed kidney in UUO-induced RF mice. The representative morphological images of the obstructed and contralateral non-obstructed kidney tissue from each group mice (**A**). Weight of kidney (**B**). Weight ratio of the obstructed/contralateral non-obstructed kidney from each group mice (**C**). All values are represented as mean ± SD (n = 10 in each group). Superscript letters a and b designate significant differences. ^a^*P* < 0.05 compared with the model group. ^b^*P* < 0.01 compared with the model group.

As shown in Fig. 2, kidney morphology was vastly altered by UUO. Compared with the normal group, the obstructed kidneys of mice in the model group presented with abnormal swelling and a markedly increased volume of renal parenchyma (Fig. 2A). The kidney weight of the obstructed side and the weight ratio of the obstructed/contralateral unobstructed kidney in the UUO model group were also significantly increased compared with those of the normal group (*P* < 0.01) (Fig. 2B,C). However, COS could observably prevent the pathological morphologic alterations induced by UUO operation (Fig. 2A). After COS or fosinopril treatment, obviously reduced trends in the kidney weight of the obstructed side and the weight ratio of the obstructed/contralateral unobstructed kidney were observed (*P* < 0.05 or *P* < 0.01) compared with those of the model group (Fig. 2B,C). More interestingly, COS showed a dose-dependent effect on the reversal of UUO-induced renal pathological changes. The data suggested that COS could mitigate the swelling and weight of the obstructed kidney in UUO-induced RF mice.

### COS improved kidney function in UUO-induced RF mice

**Figure 3:**
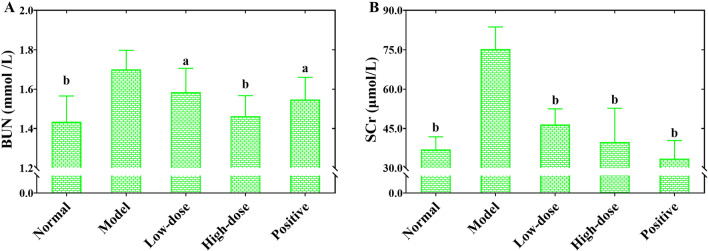
The effects of COS on the content of BUN and SCr in UUO-induced renal fibrosis mice. The concentration BUN of in serum (**A**). The concentration of SCr in serum (**B**).

As shown in Fig. 3, the contents of SCr and BUN in mice of the model group were significantly increased (*P* < 0.01) relative to those of the normal group, indicating that the normal renal function was destroyed 7 days after UUO. However, the levels of both BUN (Fig. 3A) and SCr (Fig. 3B) from the COS or fosinopril intervention group mice were significantly reduced (*P* < 0.01 or *P* < 0.05) compared to those of the model group. The results demonstrated that the renal dysfunction induced by UUO could be significantly alleviated by the treatment with COS or fosinopril. Furthermore, it is encouraging to see the dose-dependent improvement effect of COS on UUO-induced renal dysfunction. The data implied that COS can improve renal dysfunction in mice with UUO-induced RF.

### COS attenuated tubulointerstitial injury and fibrosis in UUO-induced RF mice

**Figure 4:**
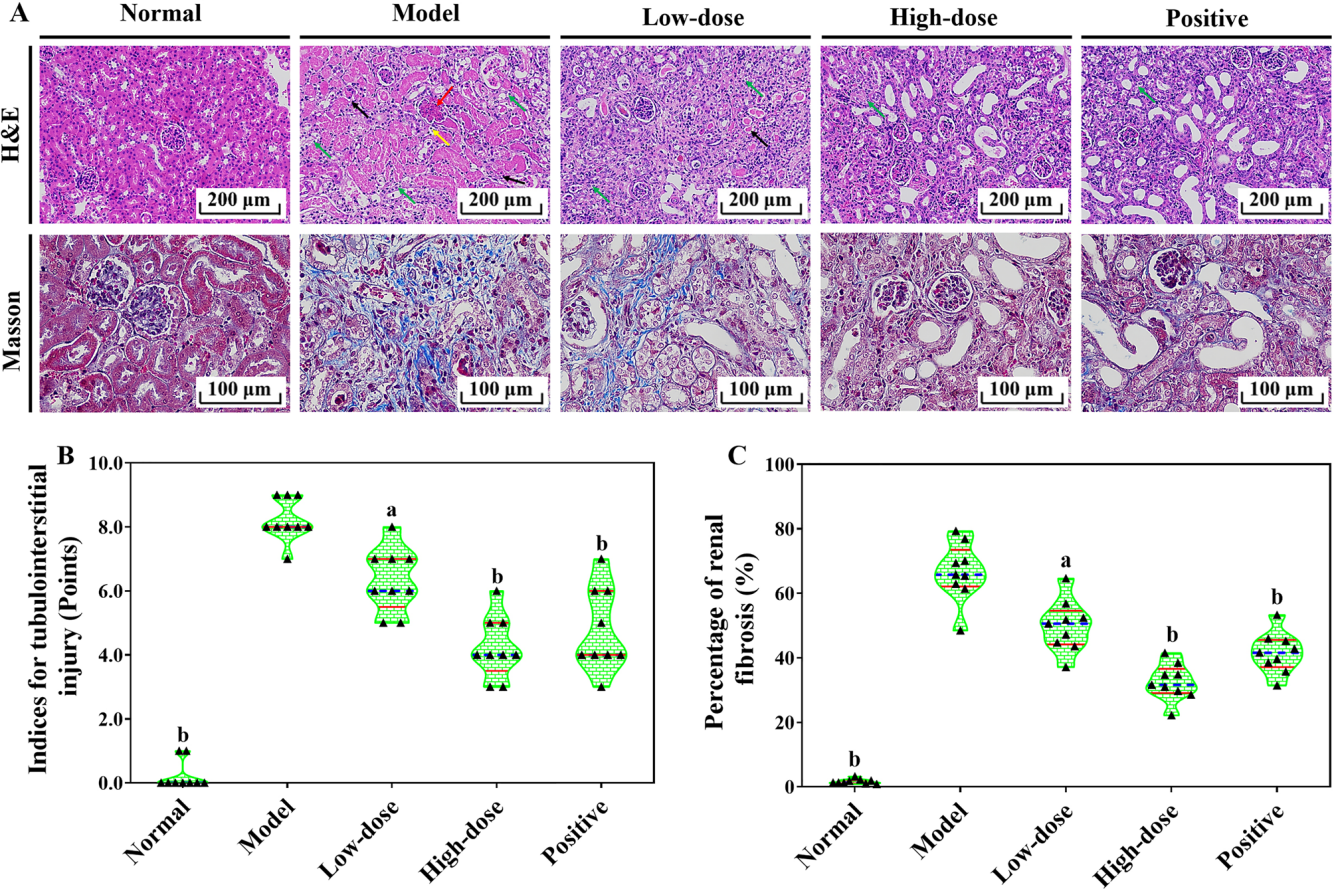
The effects of COS on the development of tubulointerstitial fibrosis in UUO-induced RF mice. Representative micrographs illustrating of H&E and Masson trichrome staining from kidney tissues (**A**). The tubulointerstitial injury score based on H&E staining (**B**). Percentage of the Masson trichrome-positive tubulointerstitial area relative to the entire area (**C**). The different colored arrows in Fig. 5A indicated the following meanings. The Black arrows implied necrosis of the tubular epithelial cells or hyperchromatism or disintegration of the nucleus or increased eosinophilic cytoplasm. The red arrows represented structural damage to the glomerulus or congestion of capillaries in the glomerulus. The yellow arrows showed inflammatory cells oozing out. The green arrows meant the epithelial cells of the renal tubules are edema and swollen, and the cytoplasm is loose, stained or vacuolar. All values are represented as mean ± SD (n = 10 in each group). Superscript letters a and b designate significant differences. ^a^*P* < 0.05 compared with the model group. ^b^*P* < 0.01 compared with the model group.

Haematoxylin and eosin (H&E) staining showed that the obstructed kidneys of mice from the model group possessed extensively dilated tubules, a large amount of tubular epithelial cell apoptosis with shedding into the lumen, and inflammatory cell infiltration (Fig. 4A). The tubulointerstitial injury indices were noticeably higher than those of the normal group (Fig. 4B). The results suggested that severe structural damage and tubulointerstitial injury were caused by UUO. At the same time, Masson trichrome staining further revealed that a large number of collagen fibers were already present in the obstructed kidney tissue of mice in the model group (Fig. 4A,C). In contrast, UUO-induced kidney obstruction was significantly reduced after the combined application of COS intervention (Fig. 4A,B), and the production of collagen fibers was notably decreased (Fig. 4A,C). Moreover, the improvement effect of COS has an obvious dose–response relationship. The key indicators for kidney injury were also summarized in Table 1. The results fully confirmed that COS can attenuate tubulointerstitial injury and fibrosis in UUO-induced RF mice.

**Table 1:** Effect of COS on alleviating kidney injury in UUO mice model.

Indexes	Treatment				
	Normal	Model	Low-dose	High-dose	Positive
Obstructed kidney	0.172 ± 0.018^b^	0.523 ± 0.168	0.343 ± 0.041^b^	0.311 ± 0.042^b^	0.306 ± 0.09^b^
Non-obstructed kidney	0.170 ± 0.016	0.185 ± 0.022	0.173 ± 0.02	0.193 ± 0.016	0.184 ± 0.017
The ratio of the obstructed/non-obstructed kidney	1.010 ± 0.072^b^	2.842 ± 0.82	1.998 ± 0.217^a^	1.622 ± 0.279^b^	1.650 ± 0.421^b^
BUN (mmol/L)	1.434 ± 0.201^b^	1.700 ± 0.097	1.485 ± 0.161^a^	1.463 ± 0.124^b^	1.547 ± 0.163^a^
SCr (μmol/L)	37.101 ± 4.760^b^	75.297 ± 8.401	46.578 ± 5.929^b^	39.914 ± 12.802^b^	33.505 ± 6.929^b^
Tubulointerstitial injury score	0.000 ± 0.000^b^	8.333 ± 0.500	6.111 ± 0.782^a^	4.222 ± 0.833^b^	4.778 ± 1.302^b^
Percentage of the Masson	1.089 ± 0.196^b^	66.744 ± 3.926	51.644 ± 3.463^a^	30.267 ± 1.733^b^	40.111 ± 1.545^b^

COS improve kidney injury in UUO mice model. All values are represented as mean ± SD (n = 10 in each group). Superscript letters a and b designate significant differences. ^a^*P* < 0.05 compared with the model group. ^b^*P* < 0.01 compared with the model group.

### COS inhibited ECM accumulation in UUO-induced RF mice

**Figure 5:**
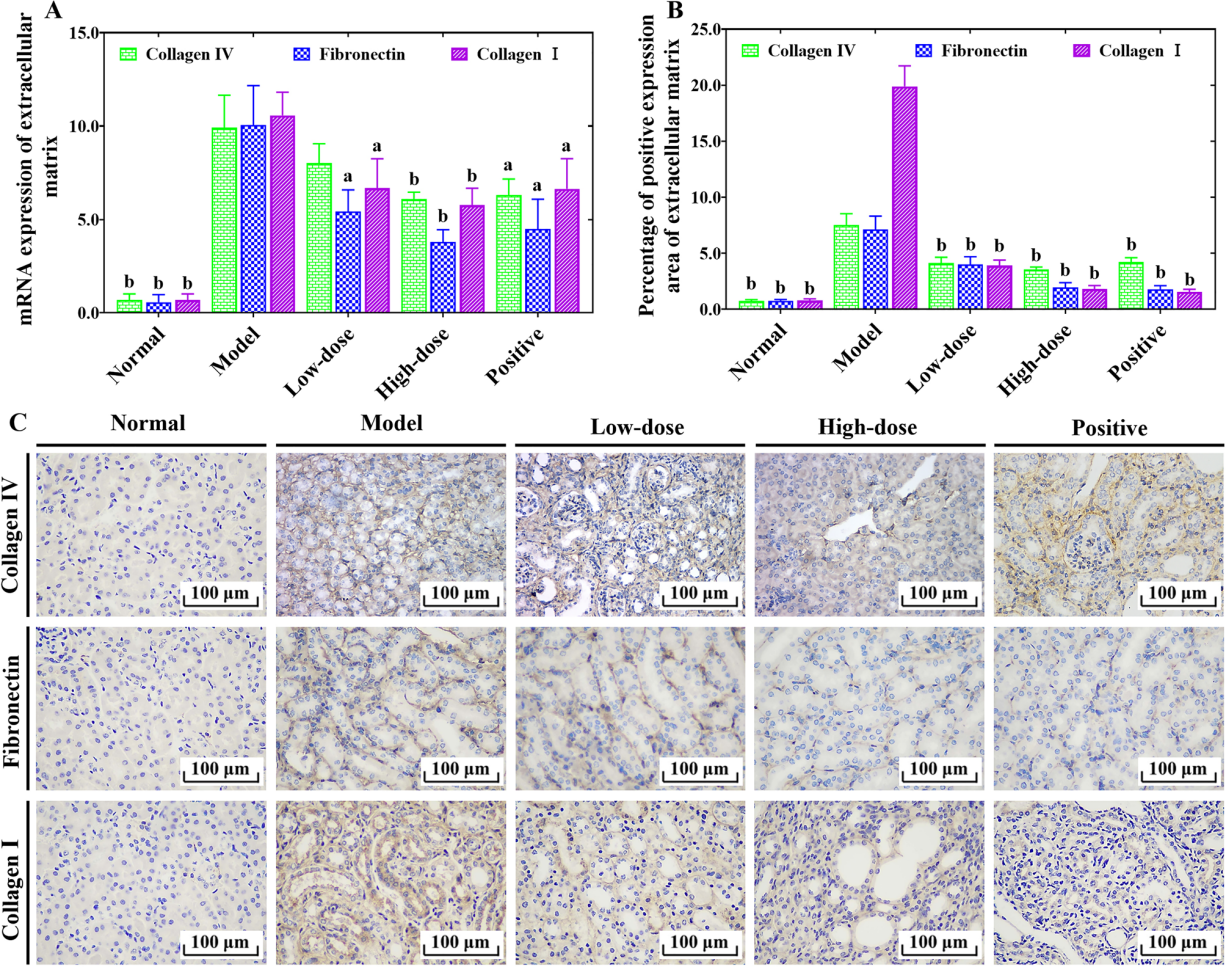
The effects of COS on ECM accumulation of kidney tissues in UUO-induced RF mice. The mRNA expression of ECM (**A**). Percentage from positive staining area of ECM to the entire field of view (**B**). Representative immunohistochemical staining image of ECM (**C**). All values are represented as mean ± SD (n = 10 in each group). Superscript letters a and b designate significant differences. ^a^*P* < 0.05 compared with the model group. ^b^*P* < 0.01 compared with the model group.

We further investigated the effect of COS on ECM in the kidney tissue of UUO-induced RF mice by examining collagen IV, fibronectin and collagen I as the representative indexes. The RT-PCR and immunohistochemical staining results showed that the expression levels of collagen IV, fibronectin and collagen I were significantly increased in the obstructed kidney tissue of mice from model group compared with that of the normal group (Fig. 5A–C). It is surprising that the excessive and abnormal collagen IV, fibronectin and collagen I secretion in obstructed kidney tissue of mice from the model group was significantly inhibited by COS and fosinopril intervention (Fig. 5). Based on these mRNA expression results (Fig. 5A) and the in situ protein expression (Fig. 5B,C), we also clearly observed that the inhibitory effect from the high-dose COS group on collagen IV, fibronectin and collagen I was obviously better than that of the low-dose of COS group. These results suggested that COS can inhibit ECM accumulation to alleviate RF.

### COS alleviated epithelial–mesenchymal transition (EMT) and inflammation in UUO-induced RF mice

**Figure 6:**
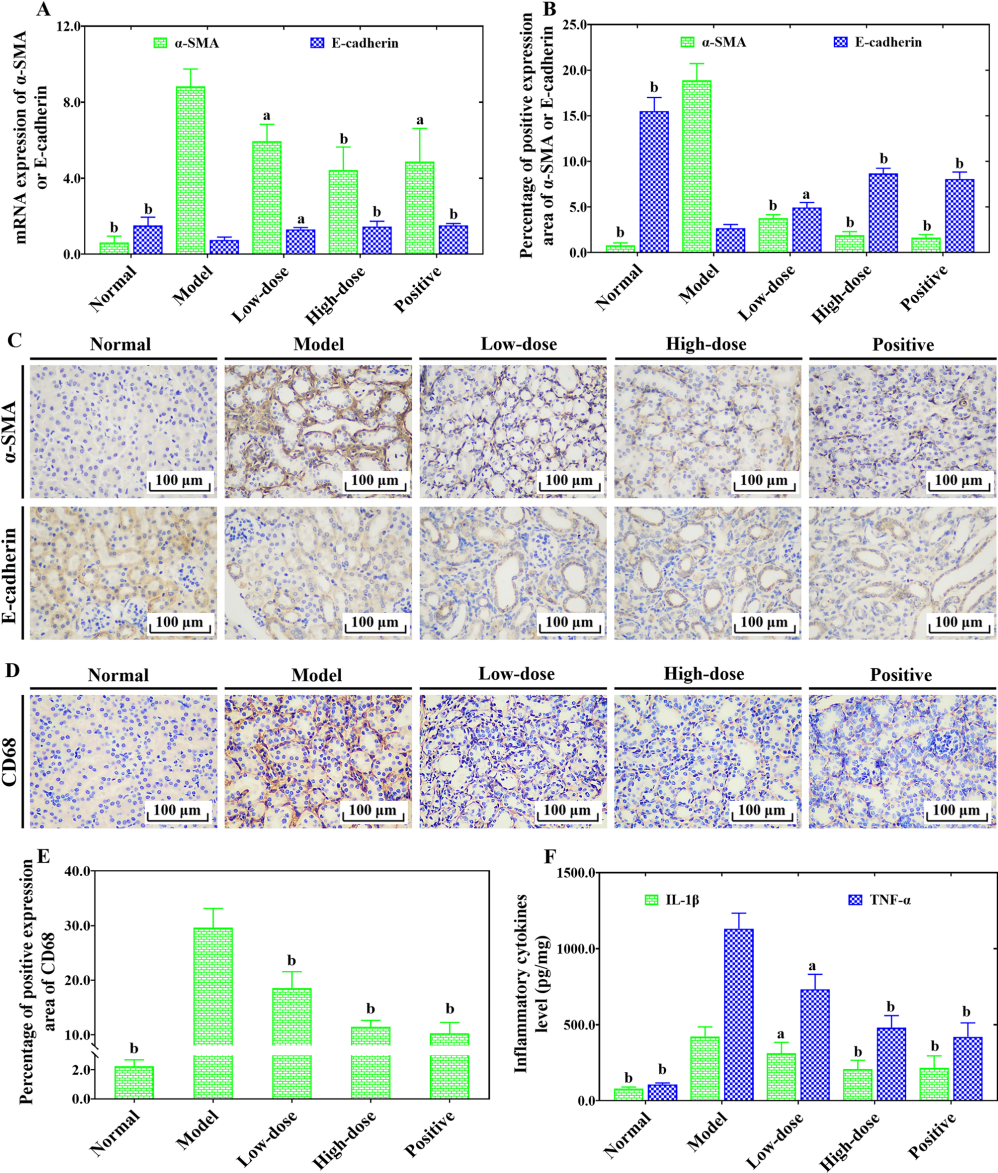
The effects of COS on α-SMA, E-cadherin and CD68 expression, and inflammatory cytokines secretion level of kidney tissues in UUO-induced renal fibrosis mice. The mRNA expression of α SMA and E-cadherin (**A**). Percentage from positive staining area of α-SMA and E-cadherin to the entire field of view (**B**). Representative immunohistochemical staining image of α-SMA and E-cadherin (**C**). Representative immunohistochemical staining image of CD68 (**D**). Percentage from positive staining area of CD68 to the entire field of view (**E**). Inflammatory cytokines secretion level of kidney tissues (**F**). All values are represented as mean ± SD (n = 10 in each group). Superscript letters a and b designate significant differences. ^a^*P* < 0.05 compared with the model group. ^b^*P* < 0.01 compared with the model group.

Elevated α-smooth muscle actin (α-SMA) and depressed E-cadherin are used as the main markers of EMT initiation^18,19^. Furthermore, the UUO-injured kidneys in mice showed massive inflammatory cell infiltration and proinflammatory cytokine secretion^18^. As one of the important markers of macrophages, CD68 expression was significantly increased in the kidneys of UUO-induced renal fibrosis model mice. As shown in Fig. 6, α-SMA expression in obstructed kidney tissue of mice from the model group was significantly elevated when compared with that of the normal group. Fortunately, the COS intervention exhibited a significant inhibitory effect on abnormal α-SMA expression as well as an elevated effect on E-cadherin expression (Fig. 6A–C). Meanwhile, we also observed that the CD68 expression and proinflammatory factor secretion in the kidneys of the model group were significantly increased compared with those of the normal group (Fig. 6D–F). The abnormal inflammatory cell invasion and secretion of proinflammatory factors in the model group were significantly inhibited by COS intervention in a dose-dependent manner (Fig. 6D–F). These results revealed that the reduction in RF by COS may be related to the inhibition of EMT and inflammation.

### COS alleviated oxidative stress injury of the obstructed kidney tissue in UUO-induced RF mice

**Figure 7:**
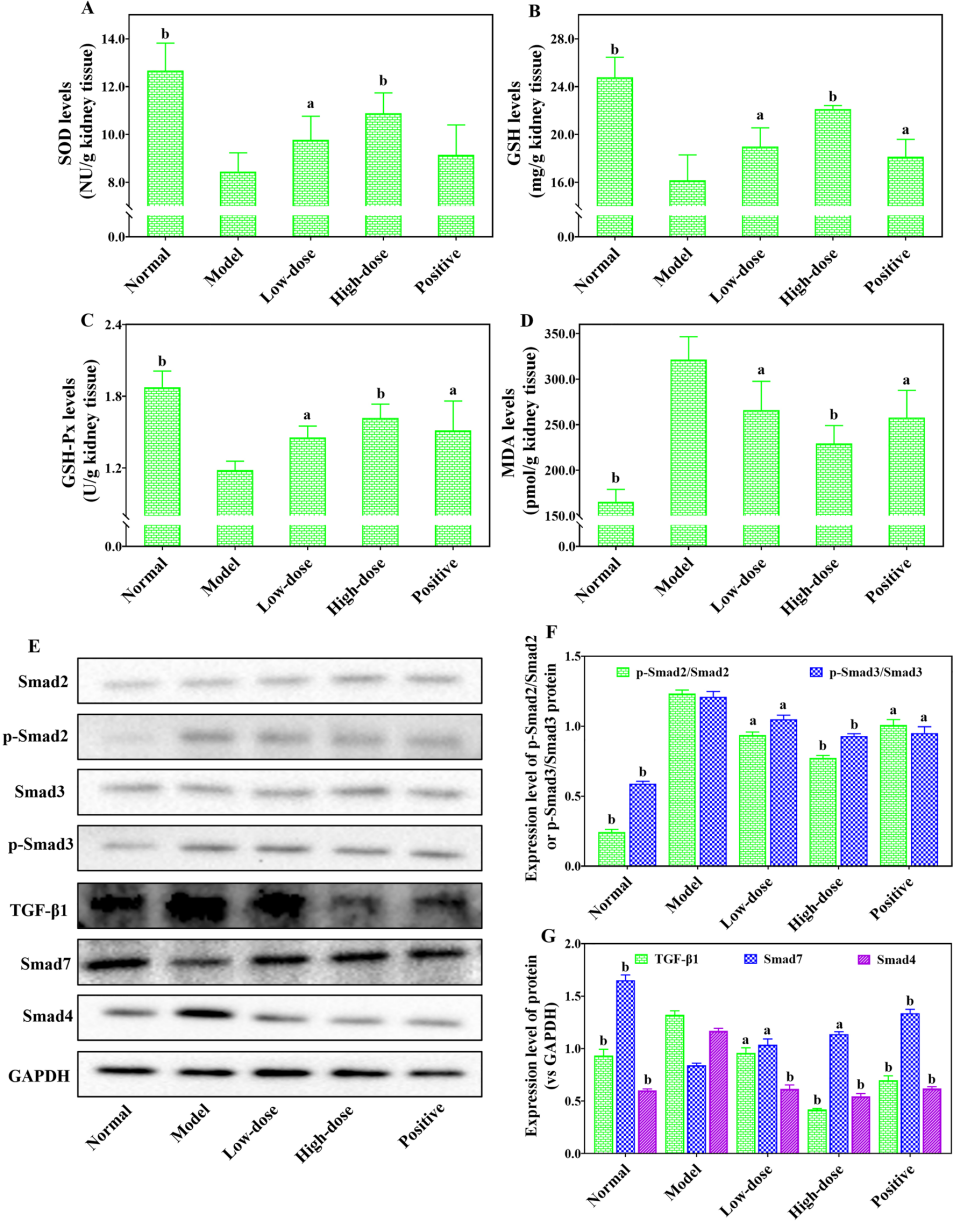
The effects of COS on the oxidative stress injury indicators and expression level of TGF-β/Smads signaling pathway protein from kidney tissues in UUO-induced renal fibrosis mice. SOD levels (**A**). GSH levels (**B**). GSH-Px levels (**C**). MDA levels (**D**). Representative Western blot analysis bands of TGF-β/Smads signaling pathway protein (**E**). Quantitative statistical chart from western blot analysis of p-Smad2/Smad2 and p-Smad3/Smad3 proteins (**F**). Quantitative statistical chart from western blot analysis of TGF-β1, Smad7 and Smad4 proteins (**G**). The original bands of Western blot from (**E**) were shown in the Supplementary Fig. S1. All values are represented as mean ± SD (n = 10 in each group). Superscript letters a and b designate significant differences. ^a^*P* < 0.05 compared with the model group. ^b^*P* < 0.01 compared with the model group.

Oxidative stress is one of the key factors in the formation of RF. Its continuous existence can stimulate a variety of cytokines and activate multiple signal pathways that lead to the occurrence and development of RF^20^. As shown in Fig. 7, the levels of SOD (Fig. 7A), GSH (Fig. 7B) and GSH-Px (Fig. 7C) in obstructed kidney tissue from model mice were significantly lower than those in the normal group, indicating that UUO induced a significant decrease in the anti-free radical ability. At the same time, a significantly higher MDA level than the normal group (Fig. 7D) also demonstrated a remarkable increase in oxygen consumption and free radical production in kidney tissue. These data suggested that the obstructed kidney tissues from model mice already had a higher state of oxidative stress injury. After COS and fosinopril intervention, however, its antioxidant capacity and free radical production were significantly enhanced and decreased, respectively. COS intervention not only increased SOD (Fig. 7A), GSH (Fig. 7B) and GSH-Px (Fig. 7C) levels, but also decreased MDA content (Fig. 7D) in the obstructed kidney tissues in a dose-dependent manner. These results confirmed that COS can prevent and cure RF by reducing oxidative stress injury.

### COS inhibited the TGF-β/Smad signal pathway in UUO-induced RF mice

A large body of evidences suggests that TGF-β plays a critical role in promoting kidney fibrosis by affecting multiple pathways and cell types, making it a prime therapeutic target^21^. Furthermore, an increasing number of studies have identified that Smad proteins as transmitters of signals in TGF-β cells^22^. Currently, dysregulation of the TGF-β/Smad pathway is an important pathogenic mechanism of RF^12^. As shown in Fig. 7E–G, the significantly upregulated TGF-β1 (Fig. 7E,G), p-Smad2/Smad2 (Fig. 7E,F), p-Smad3/Smad3 (Fig. 7E,F) and Smad4 (Fig. 7E,G) expression, as well as markedly downregulated Smad7 (Fig. 7E,G) expression, were observed in renal tissues obstructed kidney tissue of mice from the model group compared with those of the normal group. The results proved that UUO without any drug interventions could induce serious dysregulation of TGF-β/Smad pathway to promote the formation and development of RF. Amazingly, we can clearly see from Fig. 7E–G that the abnormal expression of TGF-β/Smad pathway proteins induced by UUO was significantly inhibited after the COS or fosinopril intervention. COS intervention not only attenuated the upregulation trend of TGF-β1 (Fig. 7E,G), p-Smad2/Smad2 (Fig. 7E,F), p-Smad3/Smad3 (Fig. 7E,F) and Smad4 (Fig. 7E,G) proteins induced by UUO, but also weakened the decrease in Smad7 (Fig. 7E,G) protein expression levels. More interestingly, the containment effect of COS on TGF-β/Smad pathway protein activation expression also showed an obvious dose-dependent relationship. These data fully prove that the mechanism of COS inhibition of RF may be related to its regulation of the TGF-β/Smad pathway.

## Discussion

In this study, we demonstrated that COS protected against RF in a UUO-induced mouse model. We found that COS alleviated clinical symptoms, kidney function, tubulointerstitial injury, ECM accumulation and renal fibrosis in UUO mice. Further studies indicated that COS could inhibit oxidative stress and inflammation and modulate TGF-β/Smad signal to improve renal fibrosis. More importantly, these results showed that COS might be a potential therapeutic agent for renal fibrosis.

The model of RF induced by UUO has the advantages of easy replication, reversibility, short time requirement, high success rate and small animal injury^23,24^. Currently, UUO is the most widely used model for the study of nonimmune renal tubulointerstitial fibrosis^25^. Therefore, this classic model is also used in this study to evaluate the effect of COS on alleviating renal fibrosis. The results of this study showed that the levels of BUN, SCr and the renal organ index after modeling were significantly increased. Renal hypertrophy and hyperplasia appeared in varying degrees. In addition, the kidneys of mice in the UUO model group displayed obvious pathological damage, including obvious expansion and atrophy of renal tubules, a large area of inflammatory cell infiltration, obvious focal fibrosis of the renal interstitium, and extensive shedding of the brush edge of the lumen, which were basically consistent with the UUO model lesions reported in the literature^25^. Interestingly, these indices were significantly reversed after treatment with COS or fosinopril (an angiotensin converting enzyme inhibitor that widely applied in the clinical treatment of chronic kidney disease^26^). Therefore, in this study, fosinopril was used as the reference standard, and COS has a similar effec as fosinopril.

Excessive accumulation of ECM is an important pathological basis of RF^22^. A growing body of research has also shown that continuous injury, inflammation and other factors lead to changes in the kidney tissue microenvironment, which stimulate the activation of fibroblasts and myofibroblasts to secrete a large amount of fibrotic-promoting ECM^27^. At the same time, the degradation of ECM was also inhibited. Changes in the composition, content and physical properties of the ECM lead to the shedding, apoptosis, or transdifferentiation into myofibroblasts in the kidney, which further aggravates the deposition of the ECM^28^. The normal tissue structure of the kidney is gradually compressed and eventually replaced by a dense and firm ECM, and renal function is permanently lost^19^. Interventionism against the deposition of different ECM at each stage is expected to be a potential effective means of clinical antifibrosis therapy^29^. It is exciting to see that COS could significantly inhibit the excessive and abnormal secretion of collagen IV, fibronectin and collagen I induced by UUO. In addition, a large number of studies have confirmed that when EMT occurs in renal tubular epithelial cells, the expression of E-cadherin is not only reduced or lost in renal tissues, but α-SMA will be re-expressed or increased^19^. In the present experiment, we found that the expression of α-SMA in renal tubular epithelial cells was significantly reduced by COS intervention. Moreover, E-cadherin expression was observed in most renal tubular epithelial cells. Because oxidative stress can induce an increase in reactive oxygen species (ROS) to aggravate the formation of ECM and EMT^30,31^, further studies also indicated that COS significantly improved the adverse oxidative stress injury induced by UUO. After COS intervention, increased SOD, GSH and GSH-Px secretion and the decreased MDA level compared with those of the model group were clearly observed. These results provide strong evidence confirming that COS can inhibit ECM and EMT to exhibit an excellent anti-RF effect.

Progressive RF is mediated by a variety of mechanisms and mediators, including growth factors, cytokines, metabolic toxins and stress molecules^32,33^. Among them, TGF-β1 is considered as a key factor in the pathogenesis of RF^12^. Moreover, the Smad protein is the intracellular transduction molecule of the TGF-β1 signal from the receptor to the nucleus, and it plays an important role in the regulation of signal transduction. TGF-β1/Smads belong to the most typical TGF-β signal pathway in RF regulation. When kidney injury occurs, TGF-β1 is transduced by its type I and type II threonine/serine kinase receptors on the cell membrane surface, and activates the downstream Smad2 and Smad3 proteins by phosphorylation^12^. The phosphorylated Smad2 and Smad3 proteins bind to the Smad4 protein and then shuttle to the nucleus, thereby regulating the transcription of RF related response genes^33^. In contrast, Smad7 exhibited antifibrotic effects by inhibiting the phosphorylation of Smad2 and Smad3. In this study, it was observed that the expression of TGF-β1, p-Smad2, p-Smad3 and Smad4 in the obstructed kidneys of model group mice was significantly increased except for the Smad7 reduction compared with that of the normal group, confirming the involvement of the TGF-β1/Smad signal pathway in the UUO-induced RF process. Surprisingly, COS dose-dependently inhibited the expression of TGF-β1, p-Smad2, p-Smad3 and Smad4 and increased the expression of Smad7. Their changing trends were similar to those when RF was inhibited. These results suggested that COS plays a renoprotective role in protecting against RF by inhibiting the classical TGF-β1/Smad signal pathway.

In conclusion, COS revealed a significant anti-RF effect. It can not only improve the clinical symptoms and kidney functions of RF, but also can reduce renal inflammation and structural damage and decrease ECM secretion. The improvement effect of COS on RF was closely related to the inhibition of EMT transformation and the reduction of oxidative stress injury. The anti-RF effect of COS was regulated by the TGF-β1/Smad signal pathway. Nevertheless, there were some limitations due to the experimental conditions, and the article length limit should be mentioned in the present paper. First, the current experimental evidence indicates that both COS and fosinopril are effective in treating UUO-induced RF. However, whether they have synergistic enhancement or antagonistic effects is unknown and needs to be further studied. Second, this paper was only grouped based on different doses of COS to observe its dose–effect relationship. Multiple repeated samples were used for the same COS dose intervention. Multiple cohorts need to be adopted to observe the beneficial effect of COS treatment in the future. In addition, the current experimental design used prophylactic administration and only compared the differences in kidney weight and injury between UUO and sham surgery. In further studies, the release of urinary retention should be added for systematic comparison.

## Methods

### Materials and reagents

**Figure 8:**
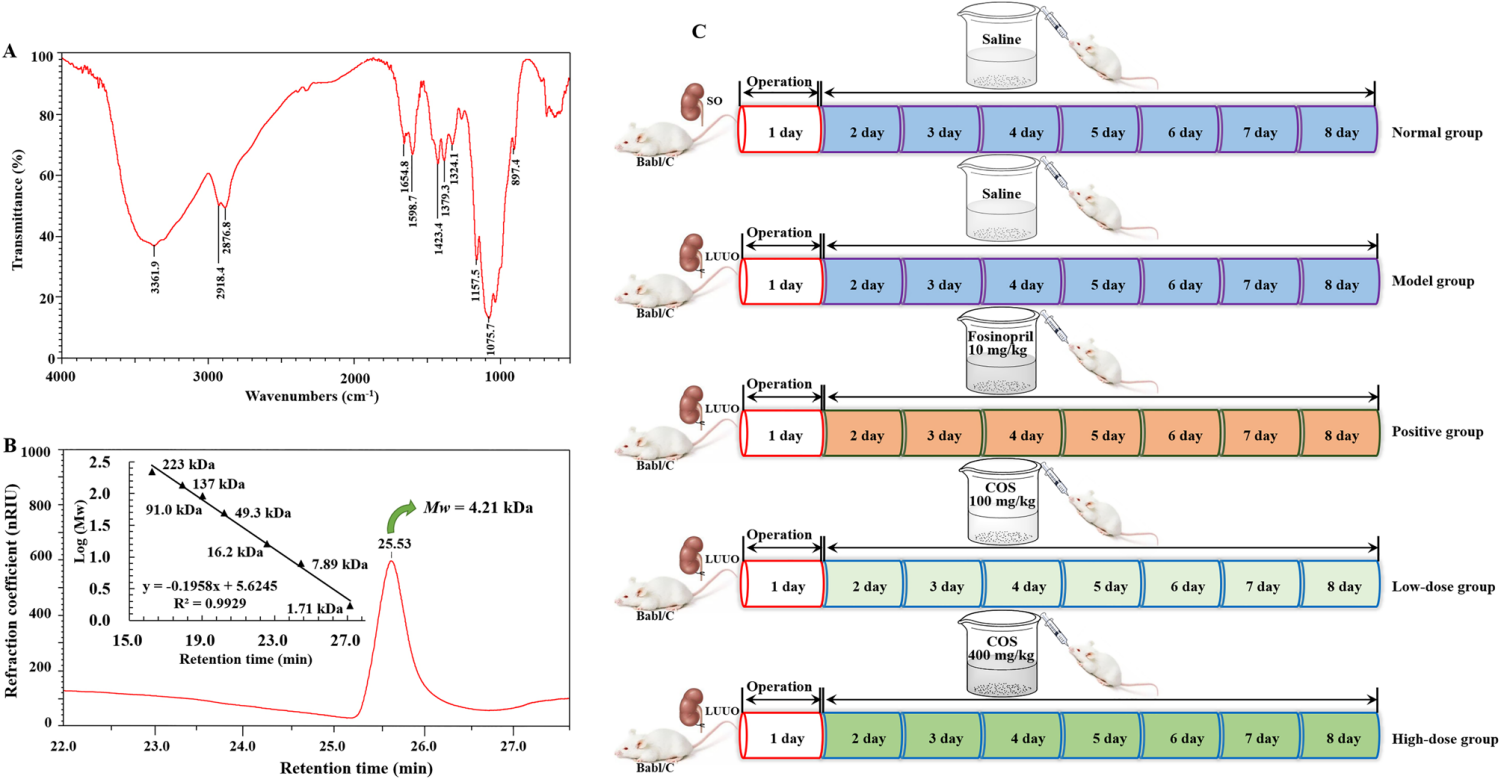
FT-IR spectrum (**A**), HPGPC and standard curve of molecular weight determination (**B**) of COS, and its intervention program design (**C**).

COS was prepared by Dr. Wu Jun in the laboratory according to the method reported^34^. In order to confirm whether the prepared sample was COS or not, it was characterized and analyzed by Fourier transform infrared spectroscopy and size-exclusion HPLC chromatography (HPGPC). As shown in Fig. 8A, the strong absorption peak at 3361.9 cm^−1^ was assigned to the stretching vibration of the –O–H and –N–H bond. The characteristic absorption peaks at 2918.4 and 2876.8 cm^−1^ were identified as the stretching vibration of C–H. Meanwhile, the deformation vibration of C–H was also found at 1423.4, 1379.3, 1324.1 cm^−1^. The 1654.8 and 1598.7 cm^−1^ were attributed to the characteristic absorption peaks of amide bond I and II. These results suggested the presence of unremoved acetyl groups in the sample. The 1157.5 and 1075.7 cm^−1^ were considered to the stretching vibration of –C–C– bond. Furthermore, the absorption peak at 897.4 cm^−1^ suggested that there was the β-d-glucosamine symmetric stretching vibration. Taking together, these characteristic absorption peaks of sample indicated that it may be COS or chitosan with a partial acetyl group. On this basis, the *Mw* of the sample was further determined to be 4.21 kDa by HPGPC (Fig. 8B). The results combined with the infrared spectrum analysis data can fully confirm that the sample used in this experiment is indeed COS. In addition, the average deacetylation degree of the sample by acid–base titration was 92.8% in accord with the results of infrared spectrum analysis.

Fosinopril was purchased from Bristol-Myers Squibb Co., Ltd. (New York, USA). Assay kits, including serum creatinine (SCr), blood urea nitrogen (BUN), SOD, GSH, GSH-Px, MDA, TNF-α and IL-1β were bought from Jiancheng Biotechnology Co., Ltd. (Nanjing, China). The primary antibodies, GAPDH primary antibodies and goat anti-rabbit secondary antibody were purchased from Abcam Co., Ltd. (Cambridge, United Kingdom). BCA Protein Assay Kit was purchased from Beyotime (Shanghai, China). ECL western blot kit was purchased from Millipore (WBKLS0500, USA). Other reagents used were of the highest commercial grade available.

### Animals and feeding

Male BALB/c mice (6–8 weeks-old and weighing 18–22 g) were purchased from the Animal Center of Guangzhou University of Chinese Medicine. Mice were placed in Experimental Animal Room (12-h light–dark cycle, 22 ± 2 °C and 60–70% relative humidity) and provided animal feeds and distilled water by free foraging. They were acclimatized to their housing environment for seven days prior to experimentation and to the experimental room for 1 h before experiments. This study was conducted strictly by the recommendations in the Guide for the Care and Use of Laboratory Animals of the National Institutes of Health. The related protocol was authorized (No. SCXK2008-0020) and supervised by the Ethics Committee for Animal Experiments of the Guangzhou University of Chinese Medicine.

### Replication of UUO model and drug intervention

The UUO model was performed by an established protocol described previously^35^. Methods were reported in accordance with the Animal Research: Reporting of in vivo Experiments (ARRIVE) guidelines. Where after, mice were randomly divided into 5 groups of Fig. 8C for the intervention. Mice in the treatment groups were administered by gastric gavage for 7 days, The doses of COS^36^ and fosinopril^37^ were used in this study according to previous reports, respectively. After the mice were anesthetized by intraperitoneal injection of 10% pentobarbital sodium solution (3 mL/kg) and blood drawn, they were sacrificed and then quickly removed their kidney tissue at the end of 8th day. The kidney tissues were carefully separated with forceps and the mucosal tissue on the surface of the organs was removed. The surface was blotted with filter paper and weighed with an analytical electronic balance.

### Renal function assessment

The level of BUN and SCr in serum were detected using corresponding assay kits. The operation procedures were in accordance with the company's instruction manual.

### Determination of inflammatory cytokines secretion levels

Determination of inflammatory cytokines secretion levels was carried out by assay kits. The operation procedures were in accordance with the company's instruction manual.

### Histopathological examination of kidney tissues

The sections of kidney tissues were stained using hematoxylin–eosin (H&E) and masson’s trichrome hematoxylin according to the reagent manufacturer's standard protocol. All histological data were obtained in a blinded manner by two independent observers. The degree of inflammation was evaluated and scored according to the method reported^38^. The degree of RF was also observed based on the area of fibrotic staining.

### Immunohistochemical analysis

The paraffin embedded sections of the kidney tissues were dewaxed in xylene, and the antigen was repaired after graded alcohol rehydrated. Sections were incubated with 3% hydrogen peroxide for 10 min to quench the endogenous peroxidase, and blocked with 5% BSA in 20 min. The diluted primary antibodies were added and incubated at 4 °C overnight. Then, the sections were incubated with a secondary antibody at 37 °C for 30 min, followed by incubation with horseradish peroxidase. DAB was used for colouring development, and hematoxylin was used to stain the nucleus. The semi-quantitative analysis of the protein expression region was performed by using image pro plus (IPP) 6.0.

### Gene expression studies by quantitative real-time PCR (qRT-PCR)

Total RNA was extracted by a High Pure RNA Isolation Kit (Tokyo, Japan), and reverse-transcribed by a Transcriptor First Strand cDNA Synthesis Kit as the manufacturer’s instructions (Roche, Germany). The PCR reaction mixture in a 20 µL volume contained 10 µL of SYBR Premix Ex Taq II (Takara Bio, Japan), 1.0 µL of reverse transcription product, 0.4 µL of sense and antisense primer sets and 8.2 µL of double distilled water. The housekeeping gene GAPDH was used as an internal standard. Supplementary Table S1 presented the primers used to amplify the genes.

### Western blot analysis

Total protein was fractionated on 8–12% SDS-PAGE gel, and then transferred to a 0.45 μm PVDF membrane. The membrane was cut into pieces based on the molecular weight of the target protein and washed three times with 1 × TBS with 0.1% Tween-20 (TBST). After incubated with 5% of non-fat milk blocking buffer, the membrane was incubated overnight at 4 °C in primary antibody. The membrane was washed in TBST three times, and then incubated in goat anti-rabbit (1:5000, ab6721, Abcam, USA), goat anti-mouse (1:5000, A21010, Abbkine, USA) or rabbit anti-goat (1:5000, A21110, Abbkine, USA) secondary antibodies for two hours. The membrane was visualized by ECL detection reagent (RPN2232, GE Healthcare, USA). Images were acquired using Tanon 6600 Luminescent Imaging Workstation (Tanon Science and Technology Co., Ltd. Shanghai, China) and quantified by ImageJ software (version 1.48v, NIH, Bethesda, MD, USA).

### Statistical analysis

Data were presented as mean ± SD unless stated otherwise. Statistical analysis for multiple groups was performed by one-way ANOVA followed by post hoc test when F achieved *P* < 0.05 and there was no significant variance in homogeneity. Some results were normalized to control to avoid unwanted sources of variation. *P* < 0.05 was considered statistically significant.

## Supplementary Information

Supplementary Information.
