# Supplementary material for: Chitosan oligosaccharide alleviates renal fibrosis through reducing oxidative stress damage and regulating TGF-β1/Smads pathway
Source: Sci Rep. 2022 Nov 10;12:19160. doi: 10.1038/s41598-022-20719-1 (PMC9649626; doi:10.1038/s41598-022-20719-1)
Supplement: Supplementary file 1 — Supplementary Information. [file 41598_2022_20719_MOESM1_ESM.docx]

**Chitosan oligosaccharide alleviates renal fibrosis through reducing oxidative stress damage and regulating TGF-β1/Smads pathway**

Jun Wu ^a^^, d^, Yingtao Xu ^a^, Zikai Geng ^c^, Jianqing Zhou ^f^, Qingping Xiong ^e^, Zhimeng Xu ^e^, Hailun Li ^b,^ *, Yun Han ^a,^ ^c,^ *

^a^ School of Chinese Medicine, Shandong College of Traditional Chinese Medicine, Yantai 264199, Shandong, PR China

^b^ Department of Nephrology, Affiliated Huai'an Hospital of Xuzhou Medical University，Huai'an 223002, Jiangsu, PR China

^c^ School of Integrated Chinese and Western Medicine, Binzhou Medical University, Yantai 264003, Shandong, PR China

^d^ Science and Technology Innovation Center, Guangzhou University of Chinese Medicine, Guangzhou, 510006, Guangdong, PR China

^e^ Jiangsu Key Laboratory of Regional Resource Exploitation and Medicinal Research, Huaiyin Institute of Technology, Huai'an 223003, Jiangsu, PR China

^f^ Department of Food, Jiangsu Food & Pharmaceutical Science College, Huai'an 223003, Jiangsu, China

*Corresponding author.

E-mail address: lihailun101@sina.com (Hailun Li); hykh888@163.com (Yun Han)

**Supplementary Table S1. Sequences of primer pairs for real-time PCR.**

| Genes | Sequences | |
| --- | --- | --- |
| GAPDH | Forward | 5-AGGAGCGAGACCCCACTAACA-3 |
|  | Reverse | 5-AGGGGGGCTAAGCAGTTGGT-3 |
| α-SMA | Forward | 5-AGGGAGTAATGGTTGGAATGGG-3 |
|  | Reverse | 5-CCTCTGTCAGCAGTGTCGGAT-3 |
| Collagen Ⅰ | Forward | 5-ATGTTCAGCTTTGTGGACCTCC-3 |
|  | Reverse | 5-GTGATACGTATTCTTCCGGGCA-3 |
| Collagen IV | Forward | 5-TATGTCCAAGGCAACGAGCGT-3 |
|  | Reverse | 5-TGCGGAATCTGAATGGTCTGAC-3 |
| Fibronectin | Forward | 5-TACACGGTTTCCCATTACGCC-3 |
|  | Reverse | 5-AGGTCTTCCCATCGTCATAGCA-3 |
| E-cadherin | Forward | 5-GGCTTCAGTTCCGAGGTCTACA-3 |

**Supplementary Table S2 The effects of COS on the clinical symptoms, weight and function of the kidneys in UUO-induced renal fibrosis (n=6)**

| **Indicators** | **Normal** | **Model** | **Low-dose** | **High-dose** | **Positive** |
| --- | --- | --- | --- | --- | --- |
| Average diet intake (g/Mouse/d) | 4.12±0.15^b^ | 3.08±0.55 | 3.48±0.37^a^ | 3.75±0.28^b^ | 3.37±0.45^a^ |
| Average water intake (g/Mouse/d) | 5.07±0.52^b^ | 3.78±0.50 | 4.23±0.45^a^ | 4.62±0.38^b^ | 4.09±0.24^a^ |
| Average body weight (g/Mouse) | 26.49±1.06^b^ | 22.33±1.84 | 24.14±1.17^a^ | 25.33±0.64^b^ | 22.79±1.11 |
| Weight of obstructed kidney (g) | 0.171±0.018^b^ | 0.523±0.107 | 0.343±0.041^b^ | 0.281±0.039^b^ | 0.306±0.091^b^ |
| Weight of contralateral non-obstructed kidney (g) | 0.171±0.016 | 0.184±0.021 | 0.172±0.019 | 0.193±0.016 | 0.184±0.017 |
| Weight ratio of the obstructed/ non-obstructed kidney | 1.009±0.072^b^ | 2.842±0.421 | 1.997±0.216^a^ | 1.454±0.278^b^ | 1.651±0.418^b^ |
| BUN (mmol /L) | 1.434±0.131^b^ | 1.711±0.097 | 1.585±0.121^a^ | 1.463±0.103^b^ | 1.547±0.113^a^ |
| SCr (μmol/L) | 37.11±4.76^b^ | 75.29±8.41 | 46.57±5.93^b^ | 39.91±12.81^b^ | 33.51±6.93^b^ |

| **Smad2** | 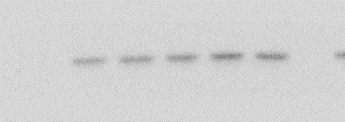 | **p-Smad2** | 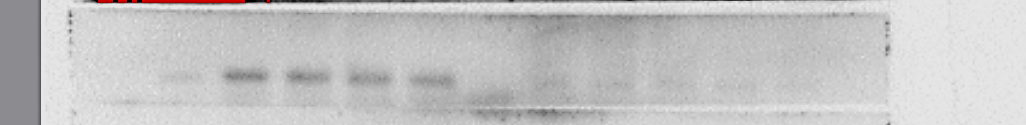 |
| --- | --- | --- | --- |
| **Smad3** | 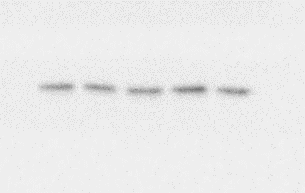 | **p-Smad3** | 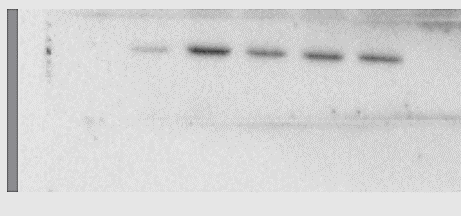 |
| **TGF-β1** | 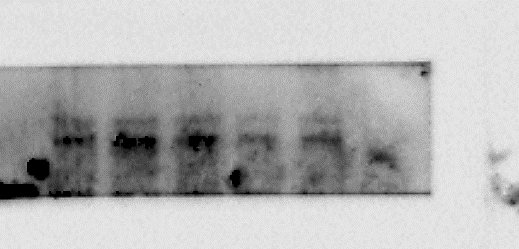 | **Smad7** | 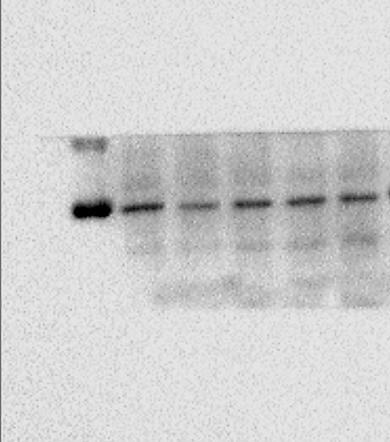 |
| **Smad4** | 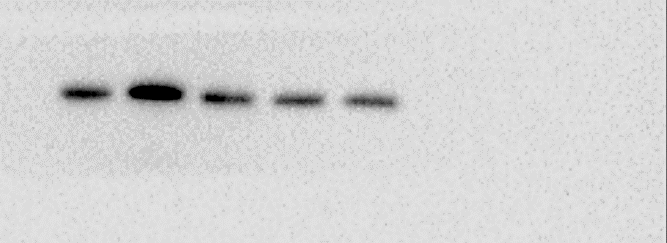 | **GAPDH** | 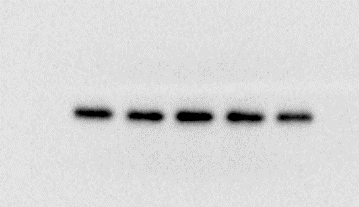 |

**Supplementary Fig. S1. The original bands of Western blot for Fig 7E.**
